# Gastrazole (JB95008), a novel CCK2/gastrin receptor antagonist, in the treatment of advanced pancreatic cancer: results from two randomised controlled trials

**DOI:** 10.1038/sj.bjc.6603058

**Published:** 2006-04-04

**Authors:** I Chau, D Cunningham, C Russell, A R Norman, T Kurzawinski, P Harper, P Harrison, G Middleton, F Daniels, T Hickish, J Prendeville, P J Ross, B Theis, R Hull, M Walker, N Shankley, B Kalindjian, G Murray, A Gillbanks, J Black

**Affiliations:** 1 Department of Medicine, Royal Marsden Hospital, Downs Road, Sutton, Surrey SM2 5PT, UK; 2 University College London Hospitals, London, UK; 3 Guy's Hospital, London, UK; 4 King's College Hospital, London, UK; 5 Royal Surrey County Hospital, Guildford, Surrey, UK; 6 Derriford Hospital, Plymouth, Dorset, UK; 7 Royal Bournemouth and Poole Hospitals, Dorset, UK; 8 James Black Foundation, London, UK; 9 University of Edinburgh, Edinburgh, UK

**Keywords:** gastrin, pancreatic cancer, fluorouracil, placebo

## Abstract

Gastrin has been shown to be a growth stimulant in pancreatic cancer cells. Gastrazole is a potent and selective gastrin receptor antagonist. Two randomised blinded trials were conducted to assess the effect of gastrazole in advanced pancreatic cancer. Patients with biopsy-proven, inoperable pancreatic carcinoma were recruited. Trial A compared protracted venous infusion (PVI) gastrazole with PVI placebo, whereas trial B compared PVI gastrazole with PVI fluorouracil (5-FU). Eighteen patients were randomised in trial A. Gastrazole produced significantly better survival compared to placebo (median 7.9 months *vs* 4.5 months; 1-year survival: 33 *vs* 11%, respectively; log rank *P*=0.02). No difference in toxicity was seen between gastrazole and placebo, except central venous catheter and pump complications. Ninety-eight patients were randomised in trial B. No significant survival difference was detected between gastrazole and 5-FU (median: 3.6 *vs* 4.2 months; 1-year survival: 13.2 vs 26.2%, respectively; log rank *P*=0.42). Toxicity of gastrazole was mild with significantly less diarrhoea (*P*=0.03), stomatitis (*P*<0.001) and hand– foot syndrome (*P*<0.001) compared to 5-FU. Quality of life (QoL) assessment showed similar QoL between gastrazole and 5-FU at baseline and no significant differences occurred with treatment either between arms or within arms. Compared to placebo, patients with advanced pancreatic cancer treated with gastrazole appeared to live longer, albeit in a very small trial and will require confirmation with large-scale randomised data. However, it did not produce survival advantage over PVI 5-FU. Lack of toxicity for gastrazole may allow its combination with cytotoxic drugs.

Pancreatic cancer accounted for approximately 232 300 cases and 227 000 deaths in 2002 worldwide (Parkin *et al*, 2005). It is associated with poor prognosis with a 5-year survival of <5%. Radical surgery is the only curative option, but only 10–15% of reported patients undergo potentially curative resection (Neoptolemos *et al*, 2003). For the remaining patients, palliative chemotherapy has been shown to improve survival and quality of life (QoL) compared to best supportive care in patients with good performance status (PS), although the survival gain was modest (Glimelius *et al*, 1996; Fung *et al*, 2003).

Gemcitabine is regarded as the current standard of care for untreated patients with advanced pancreatic cancer. The pivotal randomised controlled trial (RCT) compared gemcitabine with fluorouracil (5-FU) given over 30 min and showed that gemcitabine had a superior survival and clinical benefit response compared to short infusion 5-FU (Burris *et al*, 1997). Based on these data, the National Institute of Clinical Excellence (NICE) in the United Kingdom recommended in 2001 the use of gemcitabine monotherapy for patients with advanced pancreatic cancer. Since the pivotal study, over 5000 patients have been randomised in phase III trials comparing gemcitabine alone with other cytotoxic and biological agents either alone or in combination with gemcitabine. Gemcitabine monotherapy has consistently produced a median survival of 5–7 months and 1-year survival rate of 19–25% in these trials. Up until 2005, none of the drug combinations had shown superior results over gemcitabine monotherapy.

However, in a previously published RCT, protracted venous infusion (PVI) 5-FU produced a median survival of 5.1 months and 1-year survival of 23.5% (Maisey *et al*, 2002). Although there has not been any direct comparison between PVI 5-FU and gemcitabine, the similar survival achieved by PVI 5-FU and gemcitabine provides the rationale for the use of PVI 5-FU as a control arm in phase III studies.

In view of the poor prognosis in advanced pancreatic cancer, novel treatment strategies are required. Gastrin has been shown to be a growth stimulator in human pancreatic adenocarcinoma cells. Several studies have reported the expression of cholecystokinin (CCK) 2/gastrin receptors (both classical and an intron 4 splice variant) in human pancreatic cancer tissue (Clerc *et al*, 2002; Smith *et al*, 2002, 2004). It appears that gastrin stimulates growth of human pancreatic cancer by a receptor-mediated process and a unique CCK receptor exists in human pancreatic cancer that functions in growth, but is not found in normal human pancreas (Smith *et al*, 2002). Gastrazole (JB 95008, James Black Foundation, London) is a novel, potent and selective CCK2/gastrin receptor antagonist. *In vitro* and *in vivo* studies have demonstrated the inhibition of gastrin-stimulated growth of pancreatic cancer by gastrazole (Roberts *et al*, 2002). Because of poor (<0.1%) oral bioavailability, the drug requires administration by PVI via a central venous catheter. A pilot study was conducted in 10 patients with advanced, inoperable pancreatic cancer and their survival was compared with historical controls not undergoing active treatment (Heneghan *et al*, 2001). A significant improvement in survival was seen with gastrazole (median 7.5 months in gastrazole arm *vs* 3.3 months in historical control arm; *P*=0.03). No significant adverse events were attributed to gastrazole.

To evaluate the therapeutic effect of gastrazole in patients with advanced pancreatic cancer, two RCTs were conducted and reported here. The first study compared PVI gastrazole with placebo and was performed to assess whether the apparent survival advantage seen in the pilot study was not secondary to the additional care and attention given to patients within a clinical trial setting. In addition, despite chemotherapy, patients with advanced pancreatic cancer have a very modest survival, and for this reason, a placebo-controlled trial was considered ethically acceptable by investigators at University College Hospitals, London. The second study compared PVI gastrazole with PVI 5-FU.

## PATIENTS AND METHODS

### Study design

Both studies were designed to assess whether gastrazole would improve overall survival in patients with advanced pancreatic cancer. Trial A was a double-blinded, placebo-controlled trial in which patients were randomly allocated to receive PVI gastrazole or PVI saline/ethanol. This study was conducted in a single centre and was approved by its institutional research and ethics committee. Trial B was a single blinded trial in which patients were randomly allocated to receive PVI gastrazole or PVI 5-FU. The appearance of the drug solution (5-FU or gastrazole), dosing and all interventions were indistinguishable and were blinded to the patients. However, because of the necessary medical interventions to potential toxicity, drug treatment was not blinded to medical staff. This study was conducted in six centres in the United Kingdom and was approved by the multicentre research and ethics committee. Signed, written informed consent was obtained from each patient participating in both studies.

The primary end point in both studies was overall survival. Secondary end points included QoL, objective radiological response, toxicity, pain and analgesia requirement, weight changes and changes in serum tumour marker levels (carbohydrate antigen (CA)19-9).

### Patient eligibility

Both studies had broadly similar eligibility criteria which were: histologically or cytologically confirmed locally advanced or metastatic carcinoma of the pancreas not amenable to surgical resection or radical radiotherapy; aged 18 years old or more, adequate bone marrow, liver and renal functions with platelet >100 × 10^9^ l^−1^, white blood cell >3 × 10^9^ l^−1^, neutrophil >1.5 × 10^9^ l^−1^, bilirubin <30 *μ*mol l^−1^ (in trial B only), serum creatinine below upper limit of institutional normal range; Eastern Cooperative Oncology Group (ECOG) PS 0–2 or Karnofsky PS ⩾60%; no previous cytotoxic agent or prior radiotherapy; no significant cardiac history or alcohol abuse and life expectancy of >3 months.

### Patient randomisation

In trial A, details of all eligible patients were forwarded to a company, independent of the investigators and sponsors, who performed the randomisation based on a minimisation algorithm stratifying for tumour size (<3 cm or ⩾3 cm), presence or absence of metastases, type of intervention to palliate obstructive jaundice and/or duodenal obstruction (surgical or nonsurgical), PS, age, serum albumin and C-reactive protein. Patients were randomly assigned to each treatment arm on a 1 : 1 basis. In trial B, details of all eligible patients were forwarded to the data manager's office based at the Royal Marsden Hospital, Sutton. Eligibility criteria were verified and patients were randomly assigned to each treatment arm on a 1 : 1 basis according to a computer-generated randomisation code at an independent office. Randomisation was stratified by treatment centre, PS and presence or absence of metastases.

### Pretreatment evaluation, assessment during treatment and follow-up

Baseline evaluation included a complete medical history and physical examination, full blood count and serum biochemistry including electrolytes, liver and renal function tests, CA19-9, electrocardiogram (ECG), chest X-ray (CXR) and computed tomography (CT) of chest, abdomen and pelvis. Histological samples were reviewed by the local histopathologists and were classified into well-, moderately or poorly differentiated carcinoma. Baseline performance status, pain assessment and analgesia requirement and weight were assessed. Pain was assessed using Memorial Pain Assessment card. Quality of life was assessed using the European Organisation for Research and Treatment of Cancer core QoL questionnaire (EORTC QLQ c30).

During treatment, performance status was reassessed at each clinic visit where pain and analgesic assessment were made every 3 weeks. CA19-9, body weight and QoL were reassessed every 6 weeks. Repeat radiological assessment was made every 12 weeks. A repeat ECG was recorded after 1 week and 6 weeks of treatment only.

### Treatment

In trial A, for patients assigned to placebo, a set volume was withdrawn from a standard 0.9% sterile saline solution and an equivalent volume of absolute ethanol was replaced to achieve a concentration of 35% v v^−1^. Patients randomly assigned to gastrazole received gastrazole at 500 mg day^−1^ dissolved in saline/ethanol administered as a continuous infusion via a double lumen central venous Hickman catheter. A unit dose of 500 mg day^−1^ was used which was equivalent to 0.3 mg kg^−1^ h^−1^ for a 70 kg patient. This dose produced the highest plasma concentration that could be accommodated based on toxicity testing producing a receptor occupancy of >95% and was well tolerated in the pilot study. In trial B, patients randomly assigned to receive gastrazole would receive gastrazole at 500 mg day^−1^ identical to patients in trial A. Those patients assigned to 5-FU would receive 5-FU at 300 mg m^−2^ day^−1^ administered as a continuous infusion via a central venous catheter. No routine antiemetic medications were given. Warfarin (1 mg day^−1^ orally) was administered throughout the treatment to prevent catheter thrombosis in all patients.

Patients in trial A could continue treatment till death or intolerable toxicity, whereas patients in trial B continued treatment until evidence of radiological disease progression. All patients were followed up till death.

### Dose modifications

Toxicity was assessed according to National Cancer Institute-Common Toxicity Criteria version 2. If stomatitis, hand–foot syndrome or diarrhoea related to 5-FU developed, 50, 100 and 150 mg m^−2^ dose reductions in 5-FU were made if grades 2, 3 and 4 toxicities developed, respectively. No specific dose modification was recommended for gastrazole as no specific toxicity was anticipated.

### Evaluation of response

Radiological tumour response was evaluated according to World Health Organisation (WHO) Criteria (Miller *et al*, 1981). Complete response (CR) was defined as the complete disappearance of all measurable lesions, without the appearance of new lesion(s). Partial response (PR) was defined as a reduction of bidimensional lesions by ⩾50% of the sum of the products of the largest perpendicular diameters of each measurable lesion and no progression in other lesions or the appearance of any new lesions. Stable disease (SD) was defined as a <50% reduction of tumour volume or a <25% increase of the volume of one or more measurable lesions, with no new lesions. Progressive disease (PD) was defined as an increase of ⩾25% of the size of at least one bidimensionally measurable lesion, the appearance of new lesion(s), and/or the onset of ascites or pleural effusion with cytological confirmation.

### Role of the funding source

The investigators and representatives from James Black Foundation designed the study. The data were collected and analysed by medical and statistical representatives from Royal Marsden Hospital and James Black Foundation. All the investigators had access to the primary data and participated in writing the manuscript. All the participating institutions received grant support from James Black Foundation for conducting the study.

### Statistical considerations

In trial A, with a planned 200 patients to be randomised, it was expected that a 50% increase in median survival (from 90 days in the placebo arm to 135 days in the gastrazole arm) could be detected with at least 80% power (two-sided alpha=5%). In trial B, with a planned 172 patients to be randomised, 1-year survival improvement from 25% (5-FU arm) to 45% (gastrazole) could be detected with at least 80% power (two-sided alpha=5%).

Although both trials were planned at the same time, trial B commenced in June 1999 and trial A commenced a year later due to a delay in obtaining research and ethical committee approval. However, the accrual was slow especially towards the latter part of the trial period following the publication of NICE guidelines in 2001 when gemcitabine has become increasingly accepted as the standard treatment in the United Kingdom. In February 2003, the principal investigators in conjunction with the sponsor decided to suspend recruitment into both trials.

Tumour response rates and toxicities between treatment arms were compared using the *χ*^2^ test with Fisher's exact test used where appropriate. Serum CA19-9 level was not a normally distributed variable, thus log normalisation of CA19-9 was performed to allow parametric statistical analysis. Overall survival (OS) from randomisation was calculated from the date of randomisation into the study to the date of death from any cause. Progression-free survival (PFS) was calculated from the date of randomisation into the study to the date of either disease progression or death. Both OS and PFS were estimated using Kaplan–Meier method (Kaplan and Meier, 1958) and were compared between treatment arms using log rank test (Peto and Peto, 1972). Hazard ratios (HR) were quoted with the control arms (placebo and 5-FU) set at 1.

All analyses were performed on an intention-to-treat basis. The primary end points were updated in June 2004.

## RESULTS

**Figure 1:**
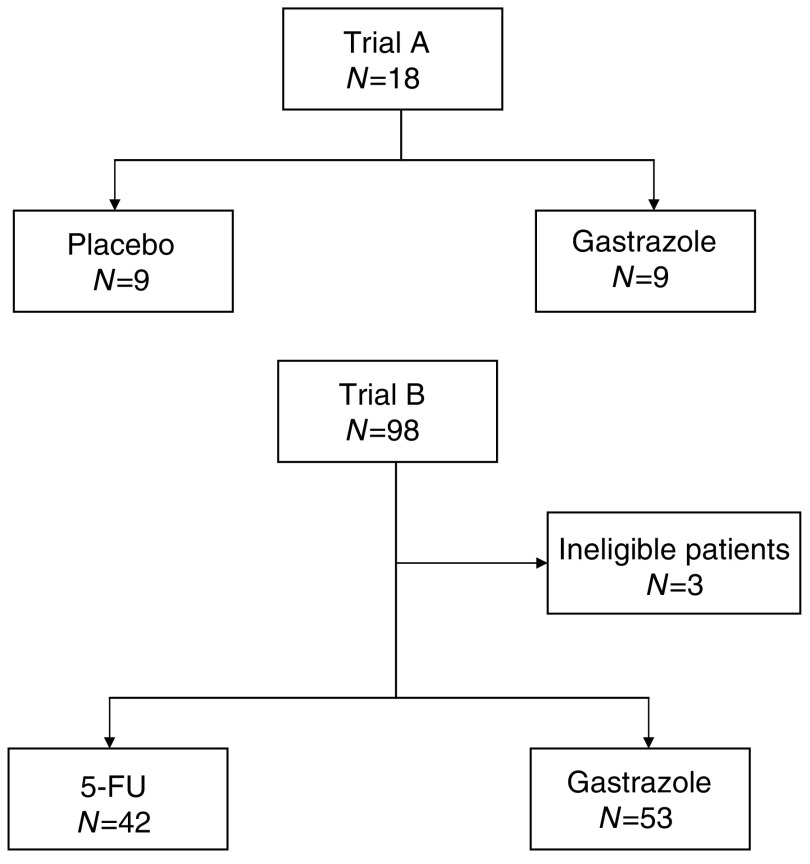
Trial profile.

Between June 2000 and February 2003, 18 patients were randomised in trial A. Between June 1999 and November 2002, 98 patients were randomised in trial B. Three patients were ineligible in trial B due to elevated serum creatinine at baseline (*n*=1), alcohol abuse (*n*=1) and no diagnosis of pancreatic cancer (*n*=1). Figure 1 shows the trial profiles. Thus, 95 patients were analysed according to an intention-to-treat basis. Table 1 shows the patients' characteristics of both trials.

### Antitumour effect

As expected, objective tumour responses were infrequent in all trial arms. No objective responses occurred in either arm in trial A. Two partial responses (5%) were seen within the 5-FU arm in trial B (Table 2). No statistically significant difference was seen between gastrazole and 5-FU (*P*=0.60).

**Figure 2:**
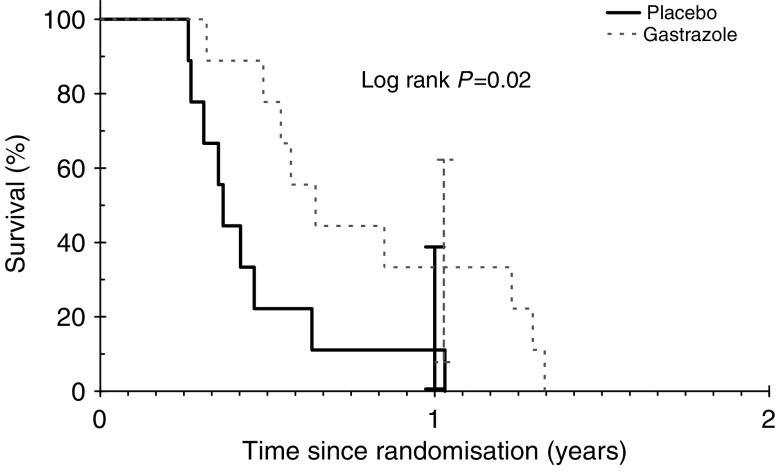
Overall survival for trial A.

**Figure 3:**
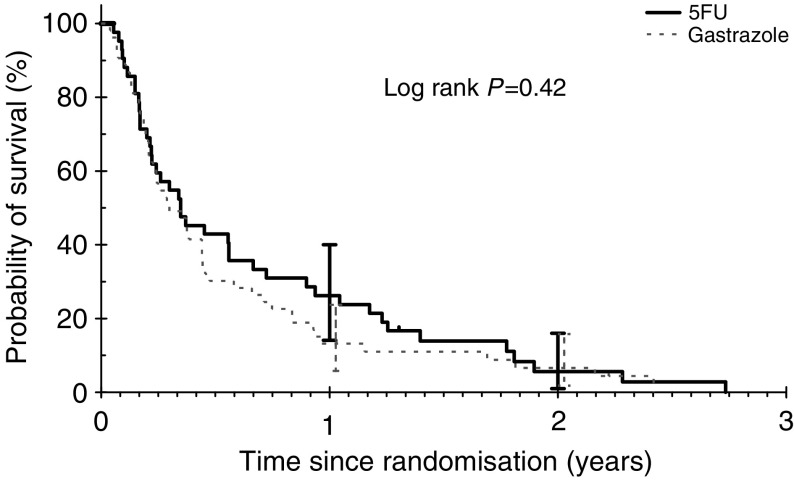
Overall survival for trial B.

At the time of analysis, all patients in trial A and 92 of the 95 (97%) patients in trial B had died. Figures 2 and 3 show the overall survival by treatment arms in trials A and B, respectively. In trial A, gastrazole was associated with a statistically significant prolonged survival (HR: 0.29; 95% confidence interval (CI): 0.10–0.85; log rank *P*=0.02). The median survival for gastrazole and placebo were 7.9 *vs* 4.5 months and the corresponding 1-year survival rates for gastrazole and placebo were 33.3% (95% CI: 7.8–62.3%) and 11.1% (95% CI: 0–38.8%), respectively. The cause of death was due to carcinoma in 17 (94%) patients.

There was no statistically significant difference in OS between gastrazole and 5-FU (HR: 1.19; 95% CI: 0.79–1.78; log rank *P*=0.42). The median survival for gastrazole and 5-FU was 3.6 months *vs* 4.2 months, respectively, and the 1-year survival rates for gastrazole and 5-FU was 13.2% (95% CI: 5.8–23.7%) *vs* 26.2% (95% CI: 14.1–40%). The causes of death were due to carcinoma in 86 (93%) patients. In trials A and B, the 60-day all-cause mortality rates were 0 and 22.1% (95% CI: 15–31.9%; gastrazole: 19%, 5-FU: 22.6%), respectively.

There were no differences in PFS between gastrazole and placebo (log rank *P*=0.56), nor between gastrazole and 5-FU (log rank *P*=0.18). The median PFS for gastrazole and 5-FU was 2.3 and 2.7 months, respectively.

### Toxicity

In trial A, toxicity was mild in both arms. Grade 3 adverse events were reported for eight patients in both the gastrazole and placebo arms and grade 4 adverse events occurred in three patients receiving gastrazole and none in those receiving placebo. However, the majority of these adverse events were considered to be disease-related rather than treatment-related. One patient developed grade 3 anaemia in gastrazole arm due to gastrointestinal bleeding unrelated to treatment, but no grade 3 or 4 neutropenia and thrombocytopenia were observed in the gastrazole arm. Deep venous thrombosis occurred in two patients in the gastrazole arm and one patient in the placebo arm. Table 3 shows the incidence of Hickman line and pump device complications by treatment arm in both trials. On visual inspection, more patients developed complications in the gastrazole arm compared to placebo arm, but no formal statistical comparisons were performed due to small number of patients in each group.

Table 4 shows the grades 3 and 4 toxicities in trial B. Toxicities commonly related to protracted administration of fluoropyrimidines such as hand–foot syndrome, stomatitis and diarrhoea were all more frequent in the 5-FU arm. There were no significant differences in the incidences of Hickman line complications between the two arms (*P*=0.07). Treatment-related mortality occurred in one patient in the 5-FU arm.

### Quality of life and clinical benefit response

In trial B, the compliance in completing QoL questionnaires was remarkably good considering the multicentre nature and the poor prognosis of the patients with less than 30% attrition rate at 6 and 12 weeks post randomisation. Table 5 shows the QoL scores in trial B. There were no significant differences in the QoL functional scores at baseline. Functional QoL was maintained in both arms during treatment with no significant differences from baseline. Patients randomised to gastrazole had significantly worse dyspnoea at baseline (*P*=0.004), but all other symptom scores were similar in both arms.

**Figure 4:**
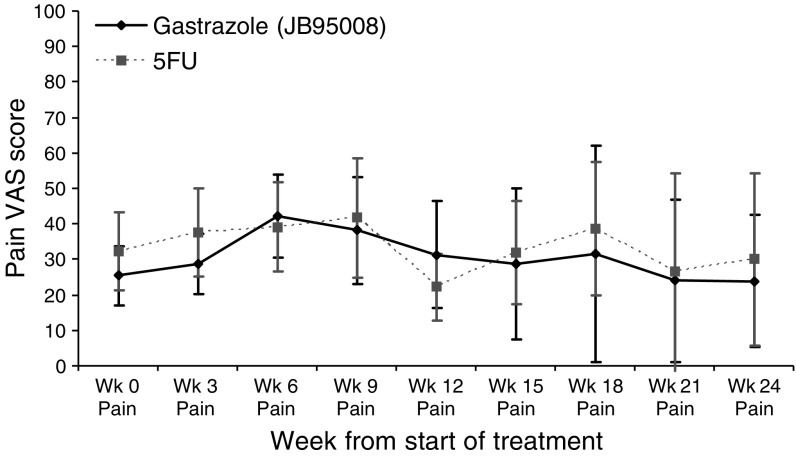
Visual analogue score for pain in trial B with 95% confidence interval. Wk: week.

**Figure 5:**
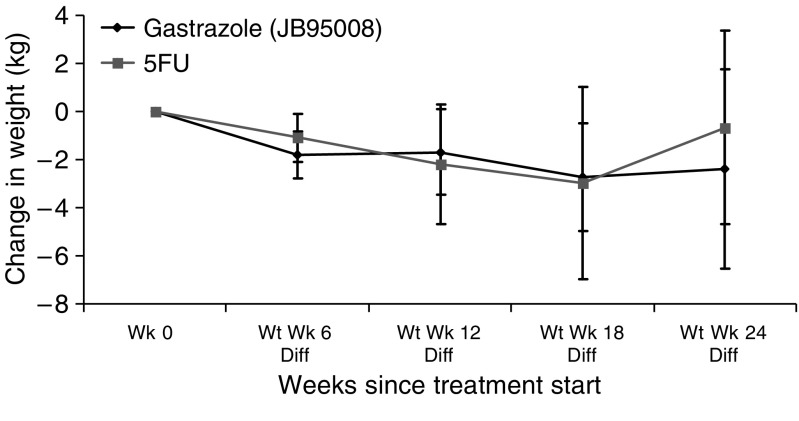
Change in weight from baseline during treatment in trial B with 95% confidence interval. Wt, weight; Wk, week; Diff, difference.

In trial B, using the visual analogue scale and pain description score, no significant differences was seen with pain between the two arms both at baseline and during treatment. Figure 4 shows these VAS scores graphically. There was no difference in the baseline weight in both arms. During treatment, there was significant weight loss in the gastrazole arm compared to baseline (*P*<0.01 at weeks 6, 12 and 18), although there was no significant difference between the treatment arms. Figure 5 shows the change in weight compared to baseline. There were no significant differences in performance status between the two arms either at baseline (*P*=0.27) or during treatment (*P*=0.55, 0.68 and 0.15 at weeks 6, 12 and 18, respectively).

**Figure 6:**
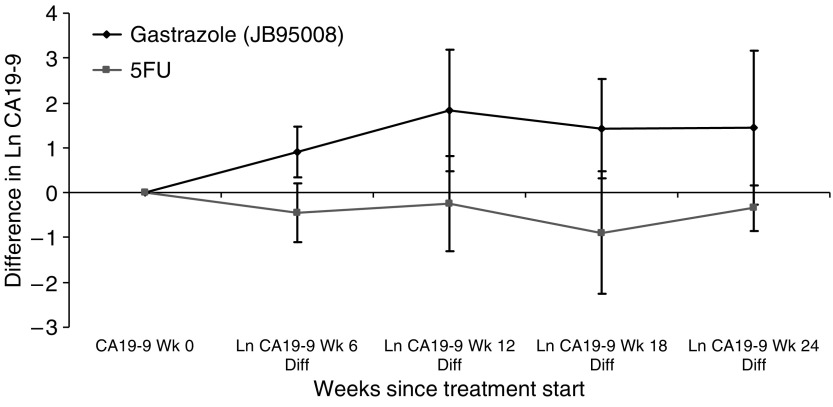
Difference in log CA19-9 level compared to baseline in trial B with 95% confidence interval. Ln, log; Diff, difference; Wk, week.

In trial A, there did not appear to be any difference in the baseline serum CA19-9 levels between gastrazole and placebo, but a much smaller increase in median serum CA19-9 levels was observed with gastrazole compared to placebo during treatment. However, no formal statistical testing was performed due to small sample size. Table 6 shows the serum CA19-9 levels in trial A. In trial B, there was no significant difference in the median serum CA19-9 level at baseline (*P*=0.54). Patients in 5-FU arm had a significant reduction in the CA19-9 levels after log normalisation (*P*<0.01 at weeks 6, 12 and 18) compared to patients in the gastrazole arm. The serum bilirubin level during the same time periods were also extracted and found to have no differences between the two arms at baseline or throughout the treatment periods. Figure 6 shows the difference in log CA19-9 level compared to baseline. Using the antilog of the log CA19-9 differences, it gave an indication of the proportional difference in the CA19-9 level from baseline. At 6 weeks, patients in the 5-FU arm experienced a 63% decrease in the CA19-9 level compared to baseline, whereas patients in the gastrazole arm experienced a 2.46 times increase (*P*<0.01).

## DISCUSSION

Advanced pancreatic cancer has attracted much research effort in recent years due to its poor outcome and lack of effective treatment. Newer cytotoxic agents such as premetrexed (Oettle *et al*, 2005), exatecan (Cheverton *et al*, 2004; O' Reilly *et al*, 2004), oxaliplatin (Louvet *et al*, 2005), irinotecan (Rocha Lima *et al*, 2004) and novel agents targeting matrix metalloproteinase (Bramhall *et al*, 2001, 2002; Moore *et al*, 2003), and farnesyltransferase (Van Cutsem *et al*, 2004) had all entered phase III testing either alone or in combination with gemcitabine – the current standard of care. Yet despite these efforts, none of these drugs have shown an OS advantage over gemcitabine monotherapy.

Targeting gastrin-induced cancer growth represents a novel therapeutic target for pancreatic cancer. *In vitro*, gastrazole alone had a significant inhibitory effect upon the gastrin-stimulated growth of BxPC-3 cells (unpublished data). The first pilot study suggested early promising survival compared to historical control (Heneghan *et al*, 2001) and this coupled with lack of toxicity of gastrazole led to the design of the two randomised studies described in this article. Although a recent meta-analysis confirmed the survival benefit for patients treated with 5-FU-based treatment compared to those who received supportive care alone (HR: 0.53; 95% CI: 0.44–0.63; *P*<0.0001) (Fung *et al*, 2003), the absolute survival gain was still very modest, thus a placebo-controlled randomised study was considered ethical in trial A. Indeed, another randomised placebo-controlled study was recently reported evaluating another gastrin-targeted strategy using G17DT, a gastrin immunogen inducing neutralising antibodies to gastrin. A borderline significant survival benefit was seen with G17DT compared to placebo (median survival 150 *vs* 83 days; Wilcoxon *P*=0.047), although there was a lower incidence of metastatic stage IV disease in the G17DT arm (73 *vs* 84%, respectively) (Gilliam *et al*, 2004). However, when G17DT was combined with gemcitabine in an RCT of 383 patients, the combination did not produce a survival advantage over gemcitabine alone (*P*=0.1) (Shapiro *et al*, 2005). In our trial A, gastrazole was associated with a longer survival compared to placebo (*P*=0.02). However, of note, there was a higher proportion of locally advanced disease in the gastrazole arm, although survival was still better with gastrazole in the subgroup of patients with locally advanced disease only compared to placebo (data not shown). The relative long survival, seen with both treatment arms in trial A was due to several reasons: firstly, the trial population was mainly consisting of locally advanced disease; secondly, this was a single institution study; thirdly and probably most importantly, the decision to continue treatment until death rather than until disease progression in this protocol. If gastrazole indeed had a tumour growth inhibitory effect, continuing treatment after radiological definition of disease progression might still had had some degree of antitumour effect, albeit small, leading to a more prolonged survival in trial A. The above reasons might have also accounted for the lower 60-day all cause mortality rates in trial A compared to trial B.

Although gemcitabine has shown survival superiority over 5-FU given over 30 min (Burris *et al*, 1997), trial B has confirmed the efficacy of PVI 5-FU in advanced pancreatic cancer to be similar to gemcitabine. The 1-year survival rates for PVI 5-FU in this study (26%) and in a previous study (24%) (Maisey *et al*, 2002) were comparable to gemcitabine (19–25%). Therefore, the use of PVI 5-FU as the control arm in this study was justified. Furthermore, QoL was maintained during treatment, which was an achievement considering the rapid deterioration of cancer-related symptoms seen commonly with advanced pancreatic cancer. Whereas the addition of bolus 5-FU to gemcitabine did not significantly improve the efficacy of gemcitabine alone (Berlin *et al*, 2002), PVI 5-FU or oral fluoropyrimidines may be a more efficacious partner to gemcitabine. Indeed, two European randomised studies evaluated the role of capecitabine in combination with gemcitabine. Both of which showed an improved efficacy with gemcitabine/capecitabine combination with the UK study showing statistically superior survival advantage over gemcitabine alone (*P*=0.026) (Cunningham *et al*, 2005). However, more recently, erlotinib, an epidermal growth factor receptor (EGFR) tyrosine kinase inhibitor, plus gemcitabine has also been reported to improve survival over gemcitabine alone (Moore *et al*, 2005).

In trial B, there was no significant difference in survival between gastrazole and 5-FU. However, because of poor accrual, the study was underpowered to show the originally proposed survival differences between the treatment and there was a hint towards better survival with PVI 5-FU. Nevertheless, no differences in QoL, symptom and clinical benefit were seen between the arms. Clinical benefit response is a composite measure of pain, analgesic use, performance status and weight loss that has been increasingly incorporated into clinical trials in metastatic pancreatic cancer. There were no significant differences in pain perception, PS and weight during treatment between the two arms. In conjunction with trial A, these data would suggest that gastrazole is biologically active. Furthermore, gastrazole was not associated with any major toxicity, and most adverse events observed during the trial were probably related to the underlying pancreatic cancer, except an increase in Hickman line and delivery device complications. Combination of cytotoxic agents with gastrazole either concomitantly or sequentially would be a feasible treatment.

Serial CA19-9 measurements have been shown to predict survival in patients treated with gemcitabine-based chemotherapy (Stemmler *et al*, 2003; Ziske *et al*, 2003) and fluorouracil-based chemoradiation (Micke *et al*, 2003). More recently, in a cohort of 154 patients treated with either PVI 5-FU, gemcitabine with or without capecitabine, patients with low baseline CA19-9 (HR: 0.56, *P*=0.0004) or 20% decrease from baseline with treatment (HR: 0.53; *P*=0.019) have been shown to have a superior survival (Maisey *et al*, 2005). CA19-9 may serve as an early indication of treatment efficacy with cytotoxic drugs, but interpretation of CA19-9 kinetics is more complex with other molecular targeted drugs. In advanced pancreatic cancer trials of marimastat, a matrix metalloproteinase inhibitor, patients with stable or falling CA19-9 level during treatment had a significantly better survival compared to those with rising CA19-9 in one trial (Evans *et al*, 2001), but not in the other (Rosemurgy *et al*, 1999). In our trial A, gastrazole appeared to slow down the increase of CA19-9 levels compared to placebo. In trial B, 5-FU was associated with a significant proportional decrease in CA19-9 whereas gastrazole showed a rising level with treatment, yet survival was not significantly different between the two arms. Cytotoxic drugs causing cell apoptosis might lead to a decrease in tumour CA19-9 secretion, whereas gastrazole, by inhibiting tumour growth, might only arrest CA19-9 increase. The relationship between CA19-9 and treatment efficacy in pancreatic cancer will need further evaluation in large randomised trials.

As gastrin is a growth stimulant, a strategy against gastrin such as gastrazole is most likely to succeed in the settings of minimal tumour burden such as maintenance therapy after cytotoxic chemotherapy (although few drugs result in significant tumour shrinkage in pancreatic cancer) or adjuvant treatment. The poor oral bioavailability of gastrazole necessitating central venous administration would not allow further clinical development of chronic administration of gastrazole in these settings, but availability of an oral gastrin receptor antagonist would facilitate the evaluation of gastrin receptor blockade in minimal disease or adjuvant settings. Moreover, the lack of serious side effects would represent a great step forward compared to bolus 5-FU/leucovorin as adjuvant treatment (Neoptolemos *et al*, 2004).

In conclusion, patients with advanced pancreatic cancer treated with gastrin receptor blockade using gastrazole appeared to live longer compared to placebo, albeit in a very small trial and will require confirmation with large-scale randomised studies. However, it did not produce survival advantage over PVI 5-FU. Lack of toxicity for gastrazole may allow its combination with cytotoxic drugs.

## Figures and Tables

**Table 1:** Baseline characteristics

**Characteristics**	**Gastrazole**	**Placebo**
*Trial A*		
Number of patients	9	9
Median age (range)	57 (44–75)	67 (55–78)
Male	6 (67%)	7 (78%)
		
*Karnofsky performance status*		
90–100%	2 (22%)	3 (33%)
70–80%	6 (67%)	6 (67%)
Unknown	1 (11%)	0 (0%)
Metastatic disease	2 (22%)	4 (44%)
		
*Tumour differentiation*		
Well	0 (0%)	0 (0%)
Moderately	3 (33%)	0 (0%)
Poorly	6 (67%)	8 (89%)
Undetermined	0 (0%)	1 (11%)
		
*Tumour subsites*		
Head	8 (89%)	7 (78%)
Body	1 (11%)	1 (11%)
Tail	0 (0%)	1 (11%)
		

**Characteristics**	**Gastrazole**	**5-FU**
*Trial B*		
Number of patients	53	42
Median age (range)	64 (35–78)	66 (38–82)
Male	27 (51%)	24 (57%)
		
*ECOG performance status*		
0	12 (23%)	7 (17%)
1	34 (64%)	26 (62%)
2	6 (11%)	8 (19%)
Unknown	1 (2%)	1 (2%)
Metastatic disease	35 (67%)	24 (59%)
		
*Tumour differentiation*		
Well	3 (6%)	4 (10%)
Moderately	24 (45%)	15 (36%)
Moderately-poorly	1 (2%)	1 (2%)
Poorly	12 (23%)	15 (36%)
Undetermined	3 (25%)	7 (17%)
		
*Tumour subsites*		
Head	40 (75%)	36 (86%)
Head+body	3 (6%)	1 (2%)
Body	3 (6%)	3 (7%)
Body+tail	2 (4%)	0 (0%)
Tail	2 (4%)	1 (2%)
Missing	3 (6%)	1 (2%)

ECOG=Eastern Cooperative Oncology Group; 5-FU=5-fluorouracil.

**Table 2:** Objective tumour response in trial B

	**Gastrazole (*n*=53)**	**5-FU (*n*=42)**	***P***
Complete response	0 (0%)	0 (0%)	
Partial response	0 (0%)	2 (5%)	
Stable disease	15 (29%)	12 (29%)	
Progressive disease	38 (71%)	28 (66%)	
Objective response rate (95% confidence interval)	0%	5% (1–16%)	0.60

5-FU=5-fluorouracil.

**Table 3:** Hickman line and pump device complications by treatment arms

	**Gastrazole (*n*=9)**	**Placebo (*n*=9)**
*Trial A*		
Infection (%)	56	22
Haematoma (%)	11	22
Device breakage (%)	33	22
Device leakage (%)	33	22
Device failure NOS (%)	78	33
		

	**Gastrazole (*n*=53)**	**5-FU (*n*=42)**
*Trial B*		
Infection (%)	21	19
Thrombosis (%)	6	0
Pneumothorax (%)	4	2
Blocked line (%)	25	2
Damaged line (%)	2	2
Any complications (%)	55	36

NOS=not otherwise specified; 5-FU=5-fluorouracil.

Category coding for Hickman line and pump device complications was different between trials A and B.

**Table 4:** Grades 3 and 4 toxicities in trial B

	**Gastrazole (*n*=53) (%)**	**5-FU (*n*=42) (%)**	***P*-values for trend**
Diarrhoea	2	7	0.03
Stomatitis	0	5	<0.001
Hand foot syndrome	0	2	<0.001
Nausea	8	5	0.76
Vomiting	9	7	0.64
Lethargy	36	33	0.95
Infection	15	10	0.34
Anaemia	6	16	0.72
Thrombocytopenia	2	3	0.82
Neutropenia	2	3	0.14

5-FU=5-fluorouracil.

**Table 5:** Quality of life scores in trial B

	**Baseline**			**6 weeks**			**12 weeks**		
	**Mean**	**s.d.**	***P*-value**	**Mean**	**Diff from base**	***P*-value**	**Mean**	**Diff from base**	***P*-value**
*Functioning scales*									

*Physical*									
Gastrazole	73.6	21.1	0.97	66.0	−6.16	0.34	66.6	−6.75	0.39
5-FU	73.3	21.3		74.1	−3.92		75.4	−0.47	
									
*Role*									
Gastrazole	53.6	35.7	0.30	51.9	−3.48	0.38	58.8	2.00	0.43
5-FU	63.1	27.8		32.2	−12.12		61.4	−10.00	
									
*Emotional*									
Gastrazole	71.1	22.4	0.76	70.6	−0.12	0.64	65.5	−0.80	0.93
5-FU	71.9	23.4		77.8	3.25		75.5	2.20	
									
*Cognitive*									
Gastrazole	78.4	19.5	0.87	73.9	0	0.96	75.1	−5.53	0.18
5-FU	78.9	19.2		79.4	−0.52		83.3	4.25	
									
*Social*									
Gastrazole	62.4	33.5	0.55	54.3	−1.88	0.08	54.2	−16.50	0.33
5-FU	69.0	25.5		56.3	−15.52		29.7	−5.25	
									
*Global*									
Gastrazole	61.4	21.9	0.42	54.5	−7.35	0.35	56.2	−8.36	0.22
5-FU	57.05	21.0		61.2	−0.33		67.7	2.00	
									
*Symptom scales*									

*Fatigue*									
Gastrazole	43.3	27.3	0.98	48.6	5.92	0.95	55.0	17.64	0.16
5-FU	42.8	23.1		43.6	6.54		38.5	4.14	
									
*N&V*									
Gastrazole	17.1	23.6	0.94	21.5	5.00	0.36	22.0	6.50	0.37
5-FU	19.3	25.8		15.6	−2.36		15.6	−2.27	
									
*Pain*									
Gastrazole	34.9	28.5	0.82	42.2	2.67	0.61	45.7	4.86	0.16
5-FU	35.6	28.5		33.9	1.27		22.9	−4.79	
									
*Dyspnoea*									
Gastrazole	21.2	25.0	<0.01	25.8	6.68	0.51	29.6	12.56	0.07
5-FU	7.0	15.8		9.5	4.93		4.1	−2.20	
									
*Sleep disturbance*									
Gastrazole	34.8	28.8	0.46	31.0	1.22	0.15	22.7	−5.56	0.33
5-FU	31.6	34.7		25.4	−10.45		10.3	22.27	
									
*Appetite*									
Gastrazole	39.9	40.6	0.67	50.6	11.26	0.99	50.8	12.94	0.09
5-FU	36.7	39.6		42.2	9.24		27.0	−4.53	
									
*Constipation*									
Gastrazole	24.2	34.0	0.24	19.8	1.27	0.28	14.6	−9.0	0.36
5-FU	31.6	32.9		17.8	−15.0		13.7	0	
									
*Diarrhoea*									
Gastrazole	15.1	25.4	0.75	17.3	1.27	0.81	26.7	11.13	0.48
5-FU	17.5	27.7		20.72	2.43		21.5	−0.06	
									
*Financial*									
Gastrazole	34.1	38.2	0.36	28.4	−7.96	0.88	24.4	−7.62	0.68
5-FU	27.9	38.1		25.6	−3.54		23.5	−10.38	

N&V=nausea and vomiting; s.d.=standard deviation; Diff from base=difference from baseline.

**Table 6:** Serum CA19-9 in trial A

	**Gastrazole (kU l^−1^)**	**Number of patients with measurement**	**Placebo (kU l^−1^)**	**Number of patient with measurement**
Baseline median level	312	8	403	9
				
*Change from baseline median*				
At 6 weeks	21	8	913	9
At 12 weeks	594	9	3234	9
At 18 weeks	739	8	50 533	4
